# Outraged Laws of Hygiene

**Published:** 1855-09

**Authors:** D. L. M’Gugin

**Affiliations:** Prof. of Physiology and Pathology in the Medical Department of the Iowa University


### OUTRAGED LAWS OF HYGIENE,

BY D. L. M'GUGIN, M. D.,

Prof. of Physiology and Pathology in the Medical Department of the Iowa University.

With what feelings of deep felt sorrow and profound regret does the medical man, who possesses, in common, extensive acquirements and a benevolent heart, look upon the multiplied and varied causes abroad in the civilized world actively at work in the physical degeneracy of our race. He looks around him and his heart is warmed with emotions of pleasure and pride when he contemplates the triumphs of genius and art and the onward advancement, steady and sure, of the march of mind, the general diffusion of intelligence, the perfection of the various systems and the multiplication of schemes of moral improvement, the happy results in the working of the benevolent enterprises for the benefit of the unfortunate of our fellow men, in fine. in almost all the schemes of human advancement toward perfection, progress is plainly written. Rejoicing in all this display of the operation of a system looking to the intellectual and moral elevation of our race, he turns around to meditate upon and deplore that much of this is unfortunately at the expense, and has been conducted antagonistic, to man's physical improvement, and which course, if persisted in, must in a few generations work an almost total degeneracy of refined and civilized nations. This regret is enhanced when he reflects that all this is wholly needless,—that with intellectual and scientific improvement, physical deterioration may be prevented;—not only so, but with intelligent direction and care the physical man could be also improved if not *pari passu* with mental and moral advancement, at least sufficiently so, to the full capabilities of force and power suggested by the wants and the spirit of progress from time to time.

But the customs and habitudes of society, as at present organized and formed, must be essentially changed to meet this design and effect this great object,—a task most discouraging to undertake, because of the difficulties which lie in the way of its accomplishment. And yet it should be undertaken, indeed it must be commenced and that too by those whose duty it is to agitate it, because they are best informed of the sad results to mankind flowing from the habits, practices, and fashions of this physically corrupt and deteriorating age. We say the medical profession owe this to philanthropy, to the best interests of the human family, to themselves, their profession, and their Creator. Who but them know aught of the nice and delicate organism of the human frame, and no class of men so well know or have so often observed, that a small and apparently trifling cause, easy of avoidance, has often sacrificed health and even life itself. We have been dealing too much in generalisms when discussing the important subject of hygiene, have not particularised sufficiently, nor have the reasons for the observance of the laws of health been urged with such force as to awaken serious alarm in the minds of those who may have had their attention partially invited to it. We must speak more boldly, more plainly, enter into particulars, and awaken in the public mind a spirit of enquiry, by suggesting fears with regard to a perseverance in many of their culpable practices. We must say most solemnly to those to whose care the young are confided that if they violate the plain dictates of reason and experience in the physical culture of children, that the God of nature will hold them to a rigid accountability if they will continue to outrage the simple laws of hygiene, thereby entailing suffering and inflicting death upon their offspring, over whom they are the guardians by nature's decree and appointment. We must say to those with whom we mingle and for whom we are required so often to prescribe for ailments plainly traceable to an open contempt for the well known and familiar rules of health, that they incur a fearful responsibility for turning nature out of her channel and contemning her manifest dictates. We must remind them too most emphatically that these physical sins, in their retribution, are not confined to themselves only, but that they do very often transfer the physical evils with their suffering consequences to others whom they bring into existence, to whom is bequeathed the dread penalty of their parents, often actually developed into activity or manifestly hanging with a fearful threatening *in terrorem* over their heads. In this way it is made to descend down and continue through several generations until proper intermarriages shall have extinguished the predisposition thus remotely engrafted, or an annihilation of the line of descent has put an end to the fearful havoc it has made. There is no limit to the dread consequences of a single infraction of nature's laws in some instances, and the responsibility and accountability will be in proportion to these unfortunate results.

We once indulged in the hope that some good would result from the interest which, a few years since, was awakened on the subject of physiology and hygiene. The whole country was traversed by itinerant lecturers and the attendance was usually large. It is true that many of these were but smatterers possessing but a surface knowledge of the subject, but yet we hoped that it would incite to further enquiry which would result in a more extended intelligence upon these subjects. We confess to a sad disappointment in this pleasing hope, for instead of profit, they have been a decided injury, as a majority of them have been the disciples and followers of some heterodox system and therefore they have really been the propagandists of empiricism wherever they have gone and have sown the seeds of medical infidelity wherever they taught.—The public mind has been tortured and warped instead of being enlightened and informed correctly or truthfully. And it is astonishing, the implicit faith reposed in these non-medical adventurers, (who were chiefly of the feminine gender,) which fact is every day illustrated. N. P. Willis practiced rigorously a hint which he doubtless borrowed from some medical authority and come out with an enrapturing account of its results. After this his box in the P. O. was crammed with letters of enquiry as to the *modus in quo* by which he was relieved, but which could be more correctly explained by their own family physician perhaps, if they would have but applied to him for his counsel. If a difficulty occurred involving some legal question of right to property or other like interest, they would have been far from applying to one who, to say the least, is as much troubled with conceit and vanity and as credulous too as any other, whose life has been spent in swelling his imagination beyond the limit of plain and simple truth, to cater for a light reading public,—who cared more for the beauty of the *dressing* than for the healthfulness of the fact or idea thus gilded and adorned.

In this way the public mind has not only been neglected, but it has been, as we have said, *mistaught* and *misled*, and it becomes the more the medical profession to enter the field of duty, wage a fierce and unrelenting warfare against false dogmas and evil practices. The journals of medicine should discuss minutely all and particular the laws of hygiene and call upon their readers to arm and equip for the work of reform. No occasion should be allowed to pass without calling the attention of those among whom they labor to the course necessary to be pursued in order to the preservation of their health, whether in eating, drinking, sleeping, apparel, exercise, bathing, or whatever else which he deems necessary to their physical well being. This voluntary service, if performed in a proper manner, and under appropriate circumstances, will be productive of much good, and will give to their patrons a ready license and freedom to propound interrogatories upon these subjects and thus the way is opened for the exercise of still further effort for good. We will call the attention of the reader to one simple fact in illustration, which falls under his observation almost every day. He is required to visit a patient whom he finds upon a bed with the curtains drawn closely around. He prescribes and leaves to return at a particular time. All this thus far is well, but has he performed his *whole* duty? We aver that he has not.—How easily he could have contrived an excuse to point out the injurious effects of those curtains, when he required them to be raised in order to afford light in the progress of investigation of the case before him. He could then have remarked that "such ornaments to the bed are objectionable, not only in excluding light, for this is sometimes desirable, but they also exclude that which is more essential to health, namely, the free circulation of pure air, absolutely required at *all* times but particularly now. To show how far a single individual will go to deteriorate the air within these curtains when drawn closely, you have only to hang your bird cage to the roof of your curtain frame, draw your curtains, and in the morning you will find that death has liberated it from confinement. This is not all. As you lie here sick and diseased, the insensible emanations which pass off from the body will be absorbed by the fabric composing the curtains, and when it becomes surcharged it will give back foul effluvia which, added to that which is continuing to pass from the surface of the body and from the lungs, will tend to perpetuate your afflictions and thwart the best efforts for cure. You would do well to remove them altogether and blind the windows if the eyes require to be shaded. For the same reason all clothing not necessary for your present comfort should be removed from your room, for they will first absorb and then reflect back the morbid matters which will deteriorate the air of the room. Beside this, you have fever and the heat of the surface is much above the natural standard. It is necessary that this excess should be conducted away as far and as speedily as possible. For this reason I have advised that the surface be spunged off with water in order that in evaporation the great heat will be conveyed away. For the same reason I have advised free ventilation, but in order to complete the work, you would do well to exchange your feather bed for a mattrass, as feathers are poor conductors. These measures will be good auxiliaries to the treatment pursued; indeed they are almost absolutely necessary."

The above is familiarly detailed in order to show how particularly we should enter into all these requisites for the well being and comfort of our patients, and as these cautions are seldom thrown away, but carefully remembered by patients as well as intelligent nurses, a fitting occasion is here presented of inculcating correct views upon this important subject of hygiene. But this is only one occasion out of a countless number of others in which, even while in the discharge of an imperative and present duty, valuable information can be imparted. And we hesitate not when we declare that however wisely, judiciously, and skillfully, the medical adviser may have prescribed therapeautic agents for the control of the disease with which from time to time he has found his patients afflicted, he has not here discharged his *whole* duty without inquiring into the necessity of hygienic precautions, and enforcing the necessity of their observance.

A common sentiment, based upon enlightened experience, is entertained against intermarriages where it is manifest that either party contain the germ in their physical system of some well known transmissible and fatal malady. Although this is a prevalent sentiment, and the practice condemned by all, yet we have no rules of public hygiene forbidding it. If we were to look forward to the physical improvement of our race, such rules would be demanded. A person knowing that there is a latent disposition to certain diseases, as insanity, consumption, scrofula, &c., without declaring the fact before marriage, such an act should be accounted fraud, and the marriage declared *null and void*. In the absence, however, of law, the medical man may do much by his timely and friendly advice, to prevent such consummation. He has seen whole families swept away, one after another—one parent or both first, then each child in turn, as they arrive at maturity. We have at this time in our thoughts a recent occurrence in which parents and several children, save one only, who died from an accident, have all died from phthisis, and the exception gave evidence of following at no distant day, parents, brothers and sisters. In this instance, beings were produced to wilt and die upon the verge of man and womanhood. They had been interwoven in the social fabric, but one thread after another parted, and the whole tissue disturbed. Hopes, gilded with bright promises, were early broken, the sacred ties of consanguinity sundered, and friendships were severed almost as soon as established. Such results inflict too much suffering upon society to be often repeated, and science, in its capacities for good, should point out the evil and warn the public mind of its unhappy consequences.

But how much more terrible are the demonstrations of this moral and physical evil, in those cases where insanity has been handed down from parent to child. Nothing can equal the intensity of the affliction where the "reason is lost in the stormy deserts of the brain." This often occurs without being able to trace any hereditary quality, but when we find, among the causes reported to the

Superintendents of Lunatic Asylums, that it is hereditary in very many cases, we are deeply impressed with the necessity of some civil regulation by which these dread results may in a good degree be prevented, and society saved from the further infliction of the direful consequences to society and to friends, to which is superadded the keen mental and physical sufferings to the individual himself. These facts should be presented to the public in every way proper, and the medical man should be diligent to disseminate correct information on this subject also. The public mind in this way can be informed, and a moral sentiment against such connexions can be so far awakened, as to be regarded as an opprobium on the character of persons when they enter into them.

The practice also of family intermarriages, and the intermingling of the same blood, is a fruitful cause of physical and mental degeneracy, and ought to be prevented by the strong arm of the law. Cousins marry, but in a large majority of instances, the effects upon the offspring are but too plainly apparent. Our experience and observation is against the practice, and from what we have seen ourselves, we are convinced it should be forbidden by statute laws upon the subject, and it is very strange that they were not long since enacted.

The degeneracy of the ruling family in England is a prominent example of this kind, but in the older States of our country, where the custom has obtained to a great extent, the results are strikingly and frequently presented. Let the young and the rising be early impressed with the horror of such connection—let it be implanted when the mind is most readily and deeply affected by impressions, and they will shudder when the partiality of parents will suggest the union of beings in whose veins there flows the same blood. Let society be taught to discourage and discountenance it and very soon the practice will be looked upon as a sacrilege.

We shall recur to this subject again, and endeavor to show that occurrences which are regarded as *providences* can be ascribed to nothing else than a suicidal contempt for the laws of hygiene, and a reckless disregard of health and life.

